# *De novo* Transcriptome Sequencing to Dissect Candidate Genes Associated with Pearl Millet-Downy Mildew (*Sclerospora graminicola* Sacc.) Interaction

**DOI:** 10.3389/fpls.2016.00847

**Published:** 2016-06-22

**Authors:** Kalyani S. Kulkarni, Harshvardhan N. Zala, Tejas C. Bosamia, Yogesh M. Shukla, Sushil Kumar, Ranbir S. Fougat, Mruduka S. Patel, Subhash Narayanan, Chaitanya G. Joshi

**Affiliations:** ^1^Department of Agricultural Biotechnology, Anand Agricultural UniversityAnand, India; ^2^Department of Biotechnology, ICAR-Indian Institute of Rice ResearchHyderabad, India; ^3^Department of Biotechnology, Junagadh Agriculture UniversityJunagadh, India; ^4^Department of Biochemistry, Anand Agricultural UniversityAnand, India; ^5^Plant Tissue Culture Lab, Anand Agricultural UniversityAnand, India; ^6^Department of Animal Biotechnology, Anand Agricultural UniversityAnand, India

**Keywords:** pearl millet, downy mildew, transcriptome, pathogenesis related proteins, hypersensitive response, phenyl propanoid pathway, SRX885597

## Abstract

Understanding the plant-pathogen interactions is of utmost importance to design strategies for minimizing the economic deficits caused by pathogens in crops. With an aim to identify genes underlying resistance to downy mildew, a major disease responsible for productivity loss in pearl millet, transcriptome analysis was performed in downy mildew resistant and susceptible genotypes upon infection and control on 454 Roche NGS platform. A total of ~685 Mb data was obtained with 1 575 290 raw reads. The raw reads were pre-processed into high-quality (HQ) reads making to ~82% with an average of 427 bases. The assembly was optimized using four assemblers *viz*. Newbler, MIRA, CLC and Trinity, out of which MIRA with a total of 14.10 Mb and 90118 transcripts proved to be the best for assembling reads. Differential expression analysis depicted 1396 and 936 and 1000 and 1591 transcripts up and down regulated in resistant inoculated/resistant control and susceptible inoculated/susceptible control respectively with a common of 3644 transcripts. The pathways for secondary metabolism, specifically the phenylpropanoid pathway was up-regulated in resistant genotype. Transcripts up-regulated as a part of defense response included classes of R genes, PR proteins, HR induced proteins and plant hormonal signaling transduction proteins. The transcripts for skp1 protein, purothionin, V type proton ATPase were found to have the highest expression in resistant genotype. Ten transcripts, selected on the basis of their involvement in defense mechanism were validated with qRT-PCR and showed positive co-relation with transcriptome data. Transcriptome analysis evoked potentials of hypersensitive response and systemic acquired resistance as possible mechanism operating in defense mechanism in pearl millet against downy mildew infection.

## Introduction

Pearl millet (*Pennisetum glaucum* (L.) R. Br.) is the sixth most important global cereal, primarily grown as a rainfed crop in the low rainfall zones of Sub-Saharan Africa and the Indian subcontinent where it contributes to the staple diet of people (Martel et al., 1997; Rajaram et al., 2013). India has seven million ha area under pearl millet with a production of 9.25 million tons (ICAR-AICPMIP Project co-ordinator review, 2015). It has wide adaptability and is looked at as one of the most significant crops in the scenario of food security and changing climate conditions. The crop productivity is severely constrained by several biotic stresses, major among them is downy mildew (DM) disease caused by the oomycete obligate pathogen, *Sclerospora graminicola* (Sacc.) Schroet. The oomycetes differ from fungi and includes economically important plant pathogens like downy mildews of poaceae, cucurbitaceae, vitaceae (Kamoun et al., 2015). The oomycetes, *S. graminicola* has been reported to hamper pearl millet yield loss up to 20–40% annually (Thakur et al., 2011). The development of downy mildew disease is favored by high relative humidity (85–90%), moderate temperature (20–30°C) and characterized by leafy inflorescence, leaf chlorosis, and failure to set seeds (Thakur et al., 2008).

The main concern regarding this oomycete is variability resulting from heterothallic nature and sexual cross compatibility among the isolates (Shetty, 1987). Besides this, the commercially released pearl millet hybrids have narrow genetic base and uniformity making it vulnerable for pathogen attack and development of new virulent strains (Thakur et al., 2011). Management strategies for the control of this disease include treatment with chemical fungicide, application of chemicals inducing resistance and resistance breeding. Of these, the most cost effective management lies in expending genetic resistance for breeding disease-resistant cultivars (Yadav et al., 2013). Moreover, genetic resistance remains a cost effective management for farmers considering the short-medium term. However, the emergence of new pathogen strains when R genes are deployed in cultivars, particularly in the case of plant-oomycete interactions (Solanum spp.-*Phytophthora* spp., Brassicaceae spp.-*Hyaloperonospora parasitica*, Poaceae *spp.-Sclerosporales*), there is a need to focus on reinforcing more sustainable resistance mechanisms (Kale, 2012; Fawke et al., 2015). Understanding such plant-oomycete interactions is vital for research interventions as it provides a way ahead to design strategies for minimizing the economic deficits caused by pathogen in crop (Dodds and Rathjen, 2010; Boyd et al., 2013). Although, the nature of pearl millet-downy mildew interaction has been studied at genetics as well as at biochemical level, there is a need to unravel the molecular basis of resistance to downy mildew infection (Thakur et al., 2011).

A comprehensive knowledge of genes is essential for undertaking genetic approaches for pearl millet improvement especially regarding disease resistance genes. The genome size of pearl millet is relatively large and current unavailability of reference genome further add to the need of identifying genes related to specific traits (Varshney et al., 2009; Varshney, 2014). In the light of this, unraveling the host-pathogen interaction and understanding the associated gene expression changes occurring at that particular time point is quintessential.

Transcriptome analysis through Next generation sequencing (NGS) technologies is a robust and efficient method for exploring the pattern of gene expression in host. Transcriptome sequencing or RNA-seq surpasses cloning and appends appreciation for the complexity of transcriptome by allowing RNA analysis through cDNA sequencing at massive scale (Margulies et al., 2005). It has been employed widely in exploiting physiological dynamics of model as well as non-model crop plants (Ozsolak and Milos, 2011). The quantification of gene expression deciphers the relative and differential expression patterns of a specific transcript or gene at a particular time point (Metzker, 2010). Transcriptome sequencing has been widely used for understanding the plant-oomycetes interaction in terms of compatibility/ incompatibility and elucidating the pathways involved in defense mechanism (Gao et al., 2013; Hayden et al., 2014; Meyer et al., 2016).

Several reports have also focussed on studying plant-pathogen interaction through transcriptome analysis (Liang et al., 2014; Reeksting et al., 2014; Weng et al., 2014; Li et al., 2015; Tan et al., 2015; Wang et al., 2015). Of more importance, the relative change in expression of host attacked by the pathogen can also be very well-tracked by RNA-seq of host (Westermann et al., 2012). In the present study, transcriptome sequencing was executed on downy mildew resistant and susceptible pearl millet genotypes upon inoculation with downy mildew pathogen and water as control in order to identify the genes and mechanism underlying downy mildew resistance.

## Materials and methods

### Pearl millet genotype and sample preparation

The seeds of downy mildew resistant genotype (P310-17) and susceptible genotype (7042S) used in the present study were obtained from the International Crop Research Institute for Semi-Arid Tropics (ICRISAT), Telangana, India. The genotypes were selected for transcriptome sequencing based on the level of downy mildew resistance and susceptibility against local Anand pathotype in the downy mildew virulence nursery (Thakur et al., 2007, 2008).

Downy mildew infected leaves of pearl millet showing whitish growth on the abaxial surface were collected in the evening from the susceptible genotype maintained in the polyhouse. The leaves were thoroughly washed with water and cleaned to remove old sporangia. Leaves were cut into small pieces, placed with abaxial surface upside in a tray lined with moistened filter paper and maintained in incubator chamber (Model I-36NL Percival Scientific Incubator, USA) at step down protocol of 20°C and 95% humidity for 8 h and at 0°C until inoculation. The sporangia were collected from the leaves showing downy mildew whitish growth using paint brush and collected in minimal required distilled water to keep the spore suspension concentrated. The inoculum was kept in dark for 15–20 min and observed under microscope for release of zoospores. Using haemocytometer, the zoospores count was adjusted to 4 × 10^4^/ml followed by inoculating the 2 days old seedlings of resistant and susceptible genotypes in three replications and water served as control (Safeeulla, 1976; Jones et al., 2001; Thakur et al., 2011). The seedlings were observed visually every 6 h for morphological changes on the surface. Seedlings harvested from three replications of each treatment were pooled after 36 h post inoculation for transcriptome sequencing. The resistant inoculated, resistant control, susceptible inoculated and susceptible control samples were represented as RI, RC, SI, and SC respectively. The methodology of experiment is represented in Supplementary Figure 1.

### 454 Sequencing

Total RNA was isolated from seedling tissues using NucleoSpin® RNA kit, Macherey-Nagel, USA following manufactures instruction. Total RNA was spectrophotometrically quantified on Infinite M200 Pro, Tecan and 1 μL of each sample was used for assessing RNA quality using RNA Nano chip on Agilent 2100 Bioanalyzer, USA. Samples having RNA Integrity Number (RIN) more than eight were processed further for mRNA isolation expending the mRNA isolation kit, Roche following manufacturer's instructions. The quality of mRNA was assessed by running each sample on RNA 6000 Pico Chip on the Agilent 2100 Bioanalyzer. The cDNA libraries were synthesized by using cDNA Rapid Library Preparation kit, Roche and assessed on an Agilent 2100 DNA High Sensitivity chip on Agilent 2100 Bioanalyzer. The cDNA libraries were clonally amplified, and sequenced on a full GS FLX Titanium Pico Titer Plate Kit 70 × 75 mm on Genome Sequencer FLX Instrument, Roche, USA available in house. Full processing was conducted, comprising both the Image Processing (i.e., “Raw wells” data files) and Signal Processing (Read flowgrams and basecalls) during the run time. The Post-Run data analysis was done after the completion of the run on a dedicated data processing computer (cluster) i.e., DataRig which is a Linux-based computer dedicated to running Genome Sequencer FLX System data processing and data analysis software, GS run processor. The sequenced raw reads were processed for removal of MID adaptor sequences by using the Perl script. Raw reads generated by 454 sequencing were deposited in the NCBI's SRA database with the accession number SRX885597.

### Pre-processing of raw reads

The raw reads emanating from sequencing of each sample were subjected to pre-processing by Prinseq-lite-0.20.4 (Schmieder and Edwards, 2011; http://prinseq.sourceforge.net/) for removal of low complexity sequences using the parameters- removal of reads with length of ≤100 bp, removal of low-quality Phred sequences of ≤20, removal of exact duplicate sequences and trimming of reads. The cleaned raw reads were further filtered for rRNA sequences using Ribopicker-0.4.3 (Schmieder et al., 2012; http://ribopicker.sourceforge.net/) with an optimized highly stringent criterion of least 90% identity and 95% coverage in BlastN search against ribosomal RNA database SILVA.

### *De novo* assembly optimization

The pre-processed high-quality reads were merged and input into the data assembly softwares *viz*., GS Assembler, Newbler (Margulies et al., 2005), proprietary software of 454 Roche with 90% identity, 40 bp overlap, cDNA option; MIRA (chevreux.org/projects_mira.html) with 454, est option in manifest file; CLC genomics workbench (www.clcbio.com) with default parameters and Trinity (Haas et al., 2013) with default parameters for assembling into transcripts. The four assemblers were evaluated based on the statistics of total number of reads used in the assembly, N50 value, largest contig size, number of contigs generated and mean contig length. For further assembly optimization, BlastX of assembled contigs was done with the close relative of pearl millet, *Setaria italica* protein database with an e-value of 1E-6.

### Functional annotation of transcripts

The assembled transcripts were BlastX with Nr database having e-value cut-off of 1E-6 on Blast2GO (Conesa et al., 2005) platform to obtain GO annotation. Blast was done against the pathogen host interaction database (PHI base) with e-value cut-off of 1E-10 (Winnenburg et al., 2006). The KEGG maps were deduced having EC numbers of the selected sequences. Graphs were plotted by WEGO, an online tool using GO numbers for functional classification (Ye et al., 2006).

### Differential gene expression analysis of transcripts

The level of transcripts expression was analyzed on the basis of number of reads mapping to each transcript. Bowtie was used for aligning the reads of each sample onto the assembled transcripts (Langmead and Salzberg, 2012). The resulting files of aligned reads were input in DESeq, a tool for quantifying the abundances of a set of target sequences from sampled subsequences based on a model using the negative binomial distribution (Anders and Huber, 2010). The differentially expressed transcripts between the pair of the samples were graphically represented by Venn diagram by inputting the transcript identifiers in VennPlex for creating Venn diagram (Cai et al., 2013). Heat maps for particular classes of transcripts were prepared using Multi experiment Viewer (MeV) v4.9.0 (Saeed et al., 2003).

### Pathway mapping of differentially expressed transcripts

The differentially expressed transcripts in resistant and susceptible genotypes were mapped to biological pathways using a web-based Kyoto Encyclopedia of Genes and Genomes (KEGG) automatic annotation server (KAAS) by executing BlastX against the manually curated KEGG GENES (Kyoto encyclopedia of genes and genomes) database. The result contains KO (KEGG Orthology) assignments and automatically generated KEGG pathways.

### Validation of prominent defense related genes through qRT-PCR

The differentially expressed transcripts involved in plant defense based on the functional annotation were selected for validation through quantitative Real-Time PCR (qRT-PCR). The fasta sequences of transcripts were retrieved and input in batch primer3 online tool (You et al., 2008) by selecting the generic option and following criterion: Product size (bp) of 100–200, primer size of 18–22 nts, temperature melting 59–62°C, rest of the parameters were default. The primer sequences of transcripts are listed in Supplementary Table 1. The first strand cDNA was synthesized from an aliquot of total RNA for each sample using RevertAid First Strand cDNA Synthesis Kit (ThermoFisher Scientific, USA) and served as template for qRT-PCR. The qRT-PCR was performed using TakaraSYBR green mix (Japan) on CFX96™ Real-Time PCR detection system, BioRad, USA following standard qRT-PCR guidelines. The stability of endogenous reference genes upon downy mildew infection in pearl millet was analyzed using RefFinder (Xie et al., 2012). RefFinder compares and rank the tested candidate reference genes based on the rankings from given by each program. Tubulin (Tub_10) was used as endogenous reference gene for normalization. The Cq values for primers were examined using CFX Manager™ software 2.1, BioRad and qBASE+ v3.0 (https://www.biogazelle.com/) was used for gene expression data analysis following Livak's –ΔΔ CT method.

## Results

**Figure 1:**
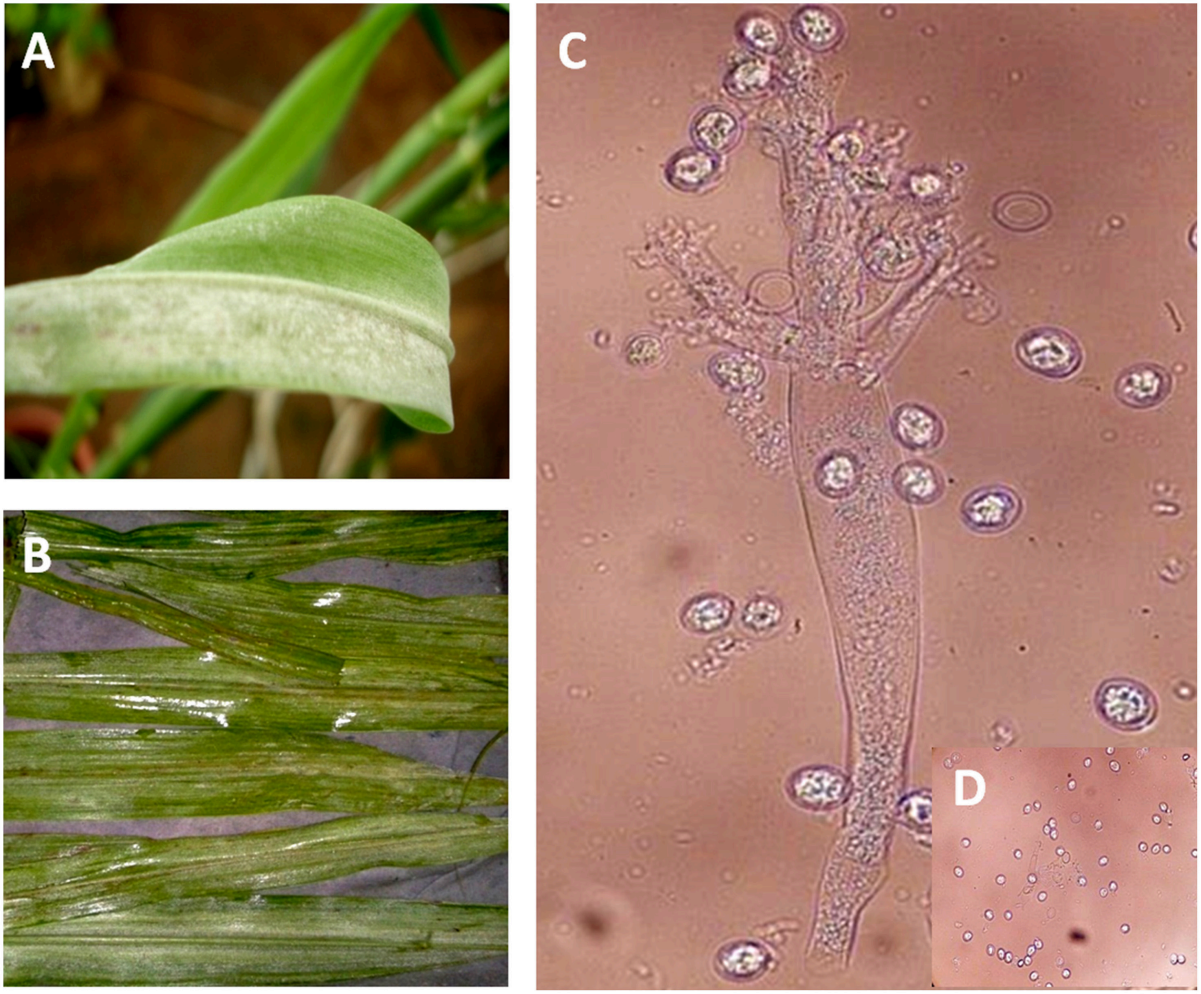
**Sporangiophore and zoospore of downy mildew pathogen. (A)** Downy mildew growth on abaxial leaf surface. **(B)** Downy mildew infected leaves kept for sporulation. **(C)** Downy mildew sporangiophore. **(D)** Zoospore release.

The downy mildew infected leaves kept for sporulation displayed whitish growth on the abaxial surface indicating profuse sporulation of the pathogen. Inoculum prepared from sporulated leaves was observed under microscopic field for its morphology and release of zoospores which is mandatory for infection. Palm shaped sporangiophore bearing sporangia; peculiar structure of downy mildew pathogen was observed (Figure 1).

### Trancriptome sequencing, *de novo* assembly, and functional annotation

The sequencing of four pearl millet libraries subjected to 454 GSFLX Titanium, Roche platform generated 684.97 Mb data. The total raw reads for the samples, RC, RI, SC, SI was 1 575 290. The pre-processing of these raw reads *viz*. low quality (0.15%), short (3.78%), duplicate (12.7%), and rRNA (0.85%) reads removal yielded a total of 1295196 high-quality (HQ) reads (~82%) with an average of 427 bases. A large percentage of HQ reads (78%) was distributed between 400 and 550 bases (Supplementary Figure 2). The detailed summary of the sequenced data and pre-processing is presented in Table 1.

**Table 1:** **Pre-processing of raw reads obtained by 454 sequencing of pearl millet genotypes (inoculated and control)**.

**Sample**	Total reads^*^	**Low-quality reads^#^**	**Trashed reads^§^**	**Duplicate reads^¶^**	**rRNA reads^†^**	High-quality reads^**^	**Average length^††^ (bases)**
RC	344,111	472	11,647	45,898	3048	283,046	432
RI	484,284	729	19,044	62,992	4055	397,464	431
SC	255,888	459	10,472	28,370	1961	214,626	424
SI	491,007	798	18,518	67,161	4470	400,060	424
Total	1,575,290	2458 (0.15%)	59,681 (3.86%)	204,421 (12.97%)	13,534 (0.85%)	1,295,196 (82.21%)	427

* *Total number of reads for each sample*.

# *Number of low-quality reads (Phred quality score of <20) removed*.

§ *Number of short reads (<100 bp) removed*.

¶ *Number of exact duplicate reads removed*.

† *Number of reads identified as rRNA sequences removed*.

** *Number of high-quality reads*.

†† *Average length of high-quality reads*.

The HQ reads were utilized for assembling into contigs, aptly known as transcripts keeping in view of transcriptome sequencing. With the increasing number of softwares available for analyzing the sequencing data, it becomes imperative to optimize the assembly for acquiring meaningful annotation of the transcripts. The detailed description of the assemblers employed and the parameters used for optimization is briefed in Table 2.

**Table 2:** **Summary statistics of ***de novo*** assembled transcripts from pearl millet genotypes (inoculated and control)**.

**Parameters/Programs**	**Newbler**	**CLC**	**MIRA**	**Trinity**
Transcripts (≥100 bases)	16658	30038	53318	26690
Total size (Mb)	13.69	24.68	42.59	25.86
Large transcripts (≥500 bases)	11005	20 299	41703	19 608
Maximum length (bases)	13594	15147	12556	17444
Average length (bases)	821	822	800	969
N50 (bases)	1099	970	838	1219
Reads mapped (%)	77.55	85.43	96.20	89.52
Number of singletons	106363	64733	36800	135425
Total size (Mb)	38.08	25.35	14.10	55.46
Transcripts with significant hits (%)^a^	12179 (73.11)	21688 (72.20)	30333 (56.89)	19986 (74.88)
Transcripts with ≥80% coverage^b^	11110	18443	25563	17356
*Setaria italica* proteins hits^c^	22213	27824	28567	27226
*S. italica* proteins with ≥80% coverage^d^	13213	18668	19679	18004

a *Transcripts showing significant hits (E ≤ 1e-6) with S. italica proteins*.

b *Transcripts showing 80% or greater coverage of S. italica proteins*.

c *Unique S. italica proteins to which transcripts show significant hits (E ≤ 1e-6)*.

d *Unique S. italica proteins to which transcripts show 80% or greater coverage*.

Out of the four assemblers, the maximum number of transcripts (53318) was registered by MIRA with total assembly size of 42.59 Mb. The number of large transcripts (≥500 bp) was higher for MIRA assembly as compared to other three assemblers. The maximum length, average length and the highest N50 value were recorded for Trinity assembler. The reads other than those utilized for generating transcripts, also known as singletons were the maximum for Trinity followed by Newbler, CLC, and MIRA. Transcripts with >80% coverage with *S. italica* proteins were represented more in MIRA as compared to the other three assemblers (Supplementary Table 2). Additionally, the percentage of HQ reads utilized for assembling by each assembler was determined by back mapping reads on to each assembly generated by individual assembler for cognizing the overall alignment rates wherein the maximum alignment percentage was shown by MIRA (96.2%) followed by Trinity, CLC and Newbler. The total number of transcripts including singletons was 90118 for MIRA. Based on all the mentioned parameters, MIRA was considered for *de novo* assembly.

**Figure 2:**
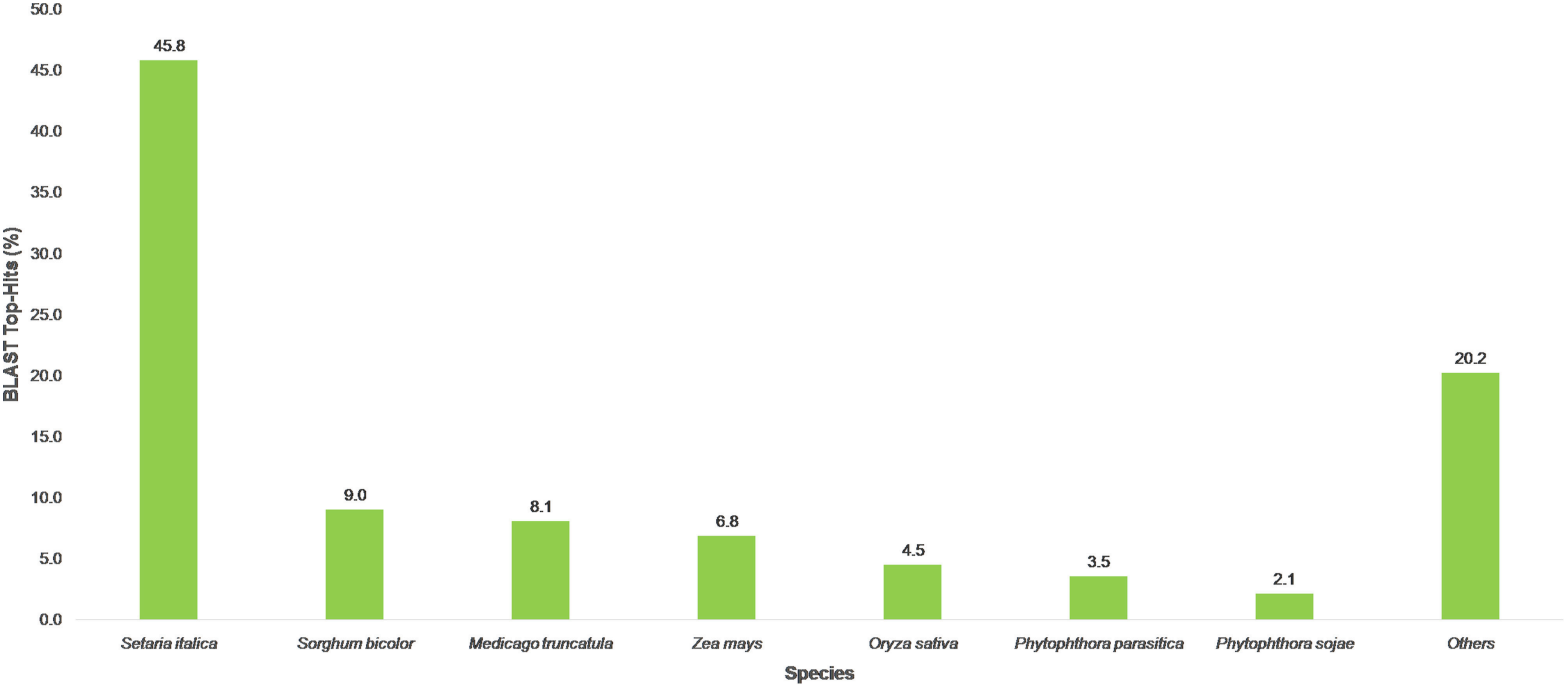
**Distribution of BLAST Top-hits species**.

**Figure 3:**
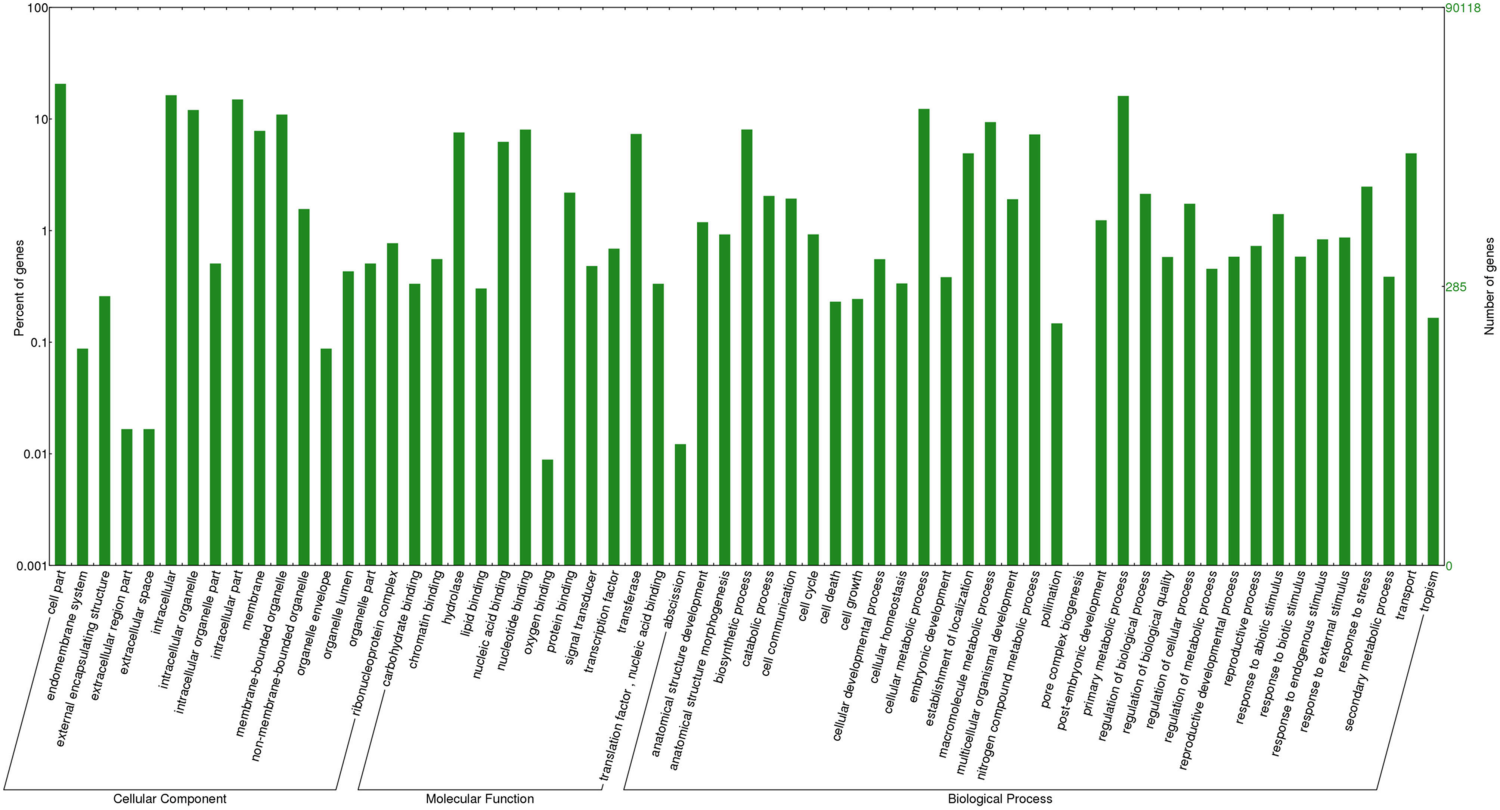
**Gene ontology distribution of pearl millet transcripts**. Percentage of transcripts categorized in cellular component, molecular function and biological process are depicted.

Out of the transcripts subjected to annotation in Blast2GO, 69% showed Blast hits and 38% of sequences were annotated against the Nr database (Supplementary Table 3). Out of the total transcripts, 7.01% were annotated as oomycetes sequences in the nr databases. The maximum number of transcripts was annotated against the UniProt Knowledgebase (UniProtKB) followed by GR_PROTEIN database and TAIR database. The annotated transcripts were subjected to KEGG pathway wherein the transcripts were linked to enzymes in a number of pathways available in KEGG. The maximum number of annotated transcripts was ascribed to hydrolases (9485) followed by transferases (4844) and oxidoreductases (2267) class of enzymes (Table 3). The data on species distribution of the transcripts conceded the highest blast hits with *S. italica* followed by *Sorghum bicolor, Medicago trunculata, Zea mays*, and *Oryza sativa* (Figure 2). Blast hits were also obtained with *Phytophthora parasitica* and *Phytophthora sojae*, two closely related oomycete species of *S. graminicola*. The pearl millet transcripts were categorized into the cellular components, molecular functions and biological processes gene ontologies (GO) and were assigned GO numbers. In cellular component ontology, the maximum number of transcripts was associated with cell (GO:0005623), intercellular organelle (GO:0043226) and membrane bound organelles (GO:0016020). In the molecular function ontology, the maximum number of transcripts were attributed to catalytic activity (GO: 0003824), binding (GO:0005488), and transporter activity (GO:0005215) while in the biological processes ontology, the maximum number of transcripts was represented by metabolic processes (GO:0008152), cellular processes (GO:0009987), and localization (GO: 0051179) (Figure 3).

**Table 3:** **Functional annotation statistics of transcripts assembled by MIRA**.

**Particular**	**# Number**
Total numbers of transcript sequences	90118
Numbers of sequences with Blast Hits	61727 (68.5%)
Numbers of sequences with Mapping	39167 (43.46%)
Numbers of sequences with Annotation	34915 (38.74%)
Total number of GOs annotation	661484
UNIPROTKB	647831 (97.94%)
GR_PROTEIN	12939 (1.96%)
TAIR	556 (0.08%)
KEGG Annotation	18484
Hydrolases	9485 (51.31%)
Transferases	4844 (26.21%)
Oxidoreductases	2267 (12.26%)
Lyases	919 (4.97%)
Ligases	638 (3.45%)
Isomerases	331 (1.79%)

### Differential expression of transcripts during pearl millet-downy mildew interaction

The 2 days old grown seedlings of resistant and susceptible genotypes inoculated with downy mildew inoculum *viz*, RI, and SI respectively, displayed hypersensitive response in the form of brown streaks which was earlier in resistant inoculated seedlings than the susceptible inoculated seedlings.

**Figure 4:**
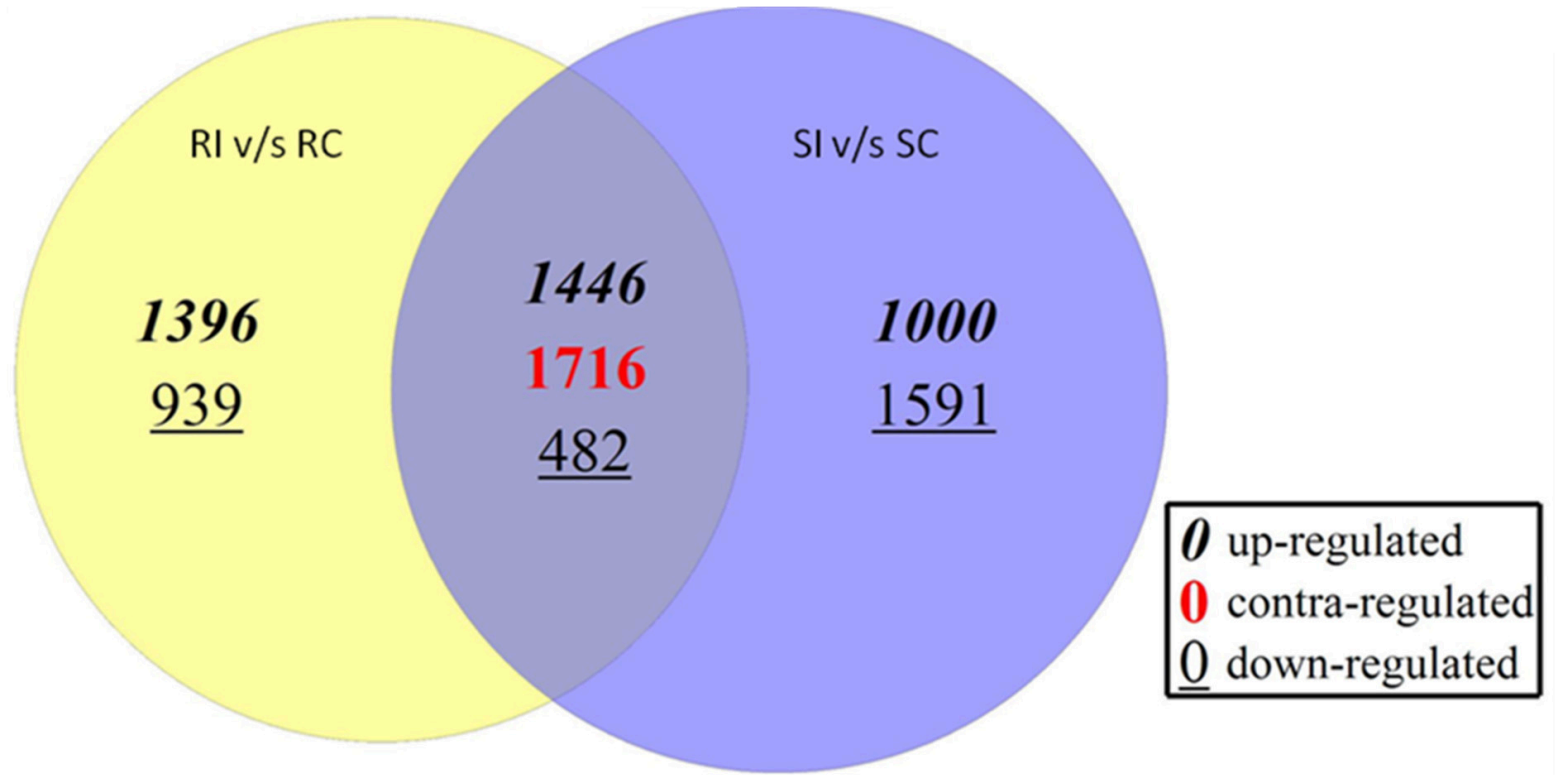
**Number of differentially expressed transcripts in resistant and susceptible pearl millet genotypes upon downy mildew inoculation**.

The observation on number of differentially expressed transcripts reflected more number of transcripts up-regulated in RI than SI. Total of 1396 transcripts were up-regulated between RI/RC and 939 were down-regulated. In the SI/SC, 1000 transcripts were up-regulated and 1591 were down-regulated. The transcripts commonly up and down regulated between RI/RC and SI/SC were 1446 and 482, respectively. The contra regulated transcripts i.e., commonly expressed but with diverse regulation polarity values were 1716 (Figure 4).

**Figure 5:**
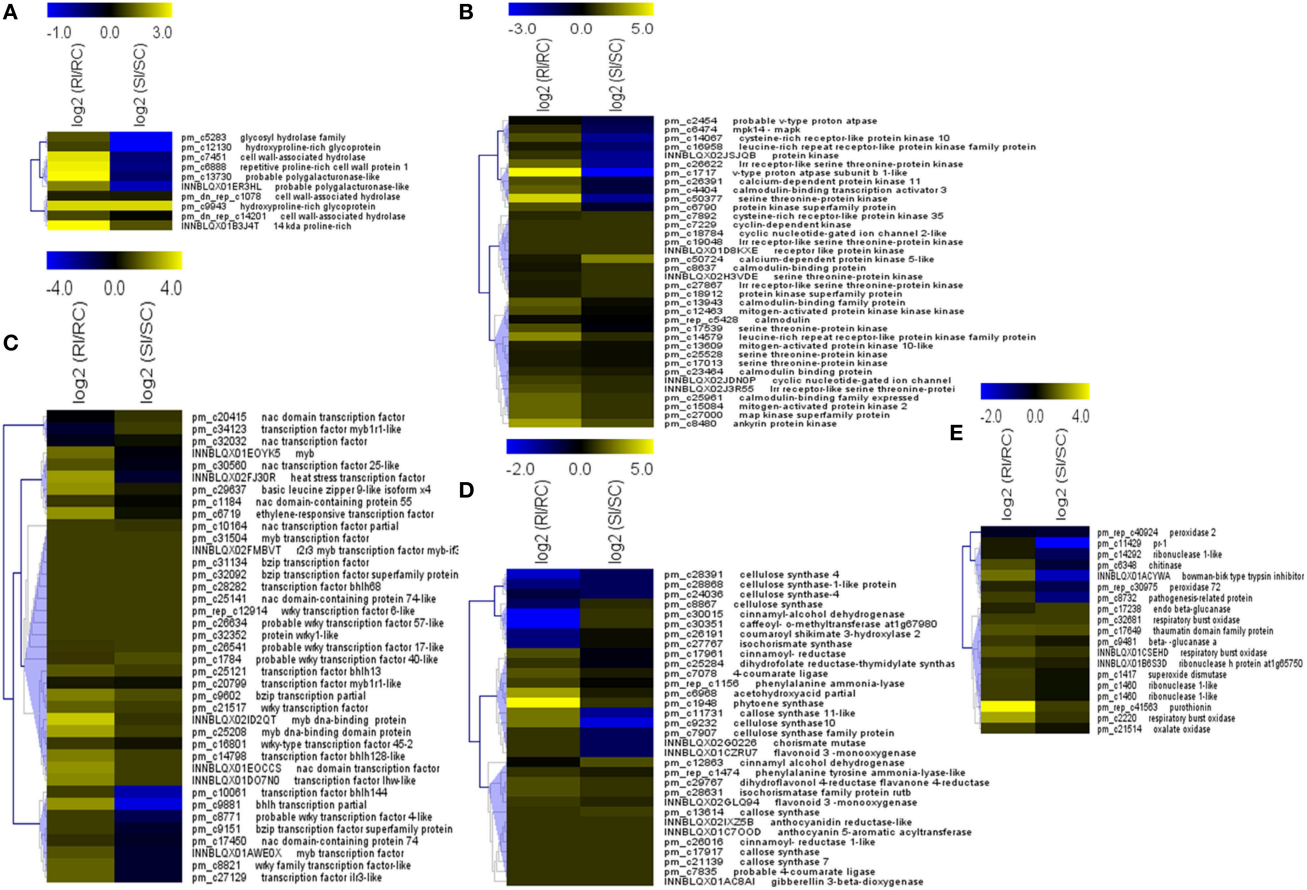
**Classes of differentially expressed transcripts in resistant and susceptible pearl millet genotypes upon downy mildew inoculation**. Transcripts for **(A)** Cell wall associated proteins, **(B)** Signaling compounds, **(C)** Transcription factors, **(D)** Secondary metabolism pathway, **(E)** Pathogenesis related proteins and hypersensitive response are depicted. Relative expression levels of each transcript is shown as heat map (hierarchical clustering with Pearson Uncentered correlation and complete linkage method). Scale represents upregulation (Yellow) to downregulation (Blue). Down regulation/Low expression 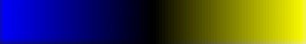 Up regulation/High expression.

Transcripts expressed as part of defense mechanism upon pathogen attack includes several classes of genes in order to render resistance to that pathogen. The response starts from the cell wall and relays *via* hormonal signaling ultimately giving resistance to the pathogen. In the present study, we divided the transcripts into classes based on their function. Differential expression was noticed for cell wall related transcripts, signaling molecules, transcription factors, and genes involved in secondary metabolic pathways (Figure 5).

The transcripts for cell wall hydrolases were differentially up-regulated with different fold changes in the resistant as compared to the susceptible. The polygalacturonase-like proteins and hydroxyproline-rich glycoprotein family proteins were also up-regulated along with exclusive expression of 14 kda proline rich proteins in resistant genotype.

In the antioxidant enzymes, respiratory burst oxidase, superoxide dismutase, and glutathione reductase were expressed differentially. One of the respiratory burst oxidase transcripts was expressed two and a half fold higher in the resistant than the susceptible genotype.

Differentially expressed transcripts involved in signal transduction included calcium signaling pathway component calmodulin, cyclic nucleotide gated channel proteins (CNGC), v-type proton ATPase, various classes of protein kinases like receptor like protein kinase, cyclic dependent nucleotide kinases (CDPK), leucine-rich repeat receptor-like protein kinase family proteins, lrr receptor-like serine threonine-protein kinases rpk2, mitogen-activated protein kinase (MAPK), mpk14, MAPKK, MAPKKK, cysteine-rich receptor-like protein kinase, and serine threonine-protein kinase. The maximum number of transcripts was attributed to serine protein threonine kinases followed by lrr receptor-like protein kinases. One transcript each for V type proton ATPase (pm_c1717), serine threonine-protein kinase (pm_c50377), ankyrin protein kinase and transcript for lrr receptor-like protein kinase (pm_c14579) were found to have relatively higher fold expression among the transcripts in resistant genotype.

The transcripts for WRKY, MYB, NAC, bZIP, bhlh transcription factor families were differentially expressed with the maximum number of transcripts for WRKY followed by NAC and MYB. Nevertheless, higher differential expression was noted for the MYB transcripts (INNBLQX02ID2QT, >3-fold change; pm_c25208, >2.5-fold change) followed by each transcript for NAC (INNBLQX01EOCCS), bhlh (pm_c9881) and bZIP (pm_c29637). One transcript each for heat stress transcription factor (INNBLQX02FJ30R) and ethylene-responsive transcription factor (pm_c6719) were also differentially expressed with higher fold change in resistant over susceptible.

Secondary metabolite pathway transcripts expressed differentially belonged to phenylpropanoid, terpenoid, and flavonoid biosynthesis pathways. The enzymes of phenylpropanoid biosynthesis pathway included phenylalanine ammonia lyase, tyrosine ammonia lyase, and chorismate mutase. Transcripts for isochorismate synthase, an enzyme for salicyclic acid precursor were highly expressed in resistant genotype. Phytoene synthase transcript was among the most highly expressed in resistant genotype. Although the expression of transcripts was noted in susceptible genotype, the level was expression was very low as compared to resistant genotype. Differentially expressed transcripts for flavonoid biosynthesis pathway enzymes included for cinnamyl alcohol dehydrogenase, flavonoid monooxygenase, coumaroyl shikimate 3-hydroxylase 2, monodehydroascorbate reductase-like, dihydroflavonol 4-reductase flavanone 4-reductase.

Total of nine classes of PR proteins were differentially represented in the resistant and susceptible genotype *viz*. pr 1, beta glucanase, chitinase, thaumatin, bowman-birk type trypsin inhibitor-like, purothionin, and oxalate oxidase. The highest expression of transcript was determined for purothionin (PR 13) in resistant genotype with four-fold higher expression. Transcripts for beta glucanase, oxalate oxidase, ribonuclease and thaumatin were also expressed in susceptible genotype but with low expression than the resistant genotype.

The differentially expressed R gene related transcripts were skp, sgt, nbs-lrr, rpp (resistance to *Peronospora parasitica*), rf45, f-box lrr, mlo, and nb-arc (Table 4). In pearl millet-downy mildew incompatible interaction, the transcripts having the highest and exclusive up-regulation were for skp protein followed by leucine-rich repeat-containing protein, disease-resistance protein sgt1nbs-lrr disease resistance protein homolog, and mlo-like protein 14. The maximum number of R gene transcripts belonged to nbs-lrr proteins, which is the largest R gene family proteins followed by rpp like proteins. Relatively low expression of R gene transcripts was noted in the susceptible genotype as compared to the resistant genotype. Transcripts for resistant gene analogs (RGA) were also up regulated in the resistant genotype. The role of RGA, belonging to non-TIR NBS LRR group has been indicated in pearl millet-downy mildew interaction (Veena et al., 2016).

**Table 4:** **Differential expression of defense related R gene transcripts**.

**Transcript ID**	**Transcript Name**	**Specifics**	**RI/RC**	**SI/SC**
pm_c22034	Disease-resistance protein sgt1	Suppressor of G2 allele of SKP1. Development of HR during R gene-mediated disease resistance	2.32	0
pm_c11050	skp1-like protein 21-like isoform x1	Component of SCF(ASK-cullin-F-box) E3 ubiquitin ligase complexes, F-box domain	4.00	0
pm_rep_c15934	skp1 interacting partner	Component of SCF(ASK-cullin-F-box) E3 ubiquitin ligase complexes, F-box domain	1.00	0.26
pm_c28289	Disease resistance protein rpm1	Nucleotide-binding, LRR HR	1.00	0
pm_c28993	Disease resistance protein rpm1	Nucleotide-binding, LRR HR	1.00	0
pm_c32590	Disease resistance protein rpm1-like	Nucleotide-binding, LRR HR	0.00	0.26
INNBLQX01AUN3U	Disease resistance protein rpm1	Nucleotide-binding, LRR HR	1.00	0
pm_c41149	Disease resistance protein rpm1	Nucleotide-binding, LRR HR	0.00	−0.74
pm_c28390	Disease resistance rpp13-like protein 1-like	Coiled coil, Leucine rich repeats, NB-ARC	1.00	0
INNBLQX01AHM5E	Disease resistance rpp13-like protein 2-like	Coiled coil, Leucine rich repeats, NB-ARC	0.42	0
INNBLQX01B7G0R	Disease resistance rpp13-like protein 3-like	Coiled coil, Leucine rich repeats, NB-ARC	0.58	0
pm_c28132	Disease resistance rpp13-like protein 3-like	Coiled coil, Leucine rich repeats, NB-ARC	−1.00	0
INNBLQX01AHDT3	Disease resistance rpp13-like protein 1	Coiled coil, Leucine rich repeats, NB-ARC	−1.00	0
INNBLQX01ENYQL	Disease resistance rpp13-like protein 2-like	Coiled coil, Leucine rich repeats, NB-ARC	1.26	0.40
INNBLQX02ITIJ1	Disease resistance rpp13-like protein 1-like isoform x2	Coiled coil, Leucine rich repeats, NB-ARC	1.58	0
INNBLQX01CNUJ5	Disease resistance rpp13-like protein 2	Coiled coil, Leucine rich repeats, NB-ARC	−1.00	0
INNBLQX01B7G0R	Disease resistance rpp13-like protein 3-like	Coiled coil, Leucine rich repeats, NB-ARC	0	−1.74
INNBLQX02ITIJ1	Disease resistance rpp13-like protein 1-like isoform x2	Coiled coil, Leucine rich repeats, NB-ARC	0	−0.74
pm_c19464	Disease resistance rpp13-like protein 4	Coiled coil, Leucine rich repeats, NB-ARC	0	−1.74
pm_c15317	Disease resistance protein rpp13-like isoform x1	Coiled coil, Leucine rich repeats, NB-ARC	0	−0.74
pm_c34958	Disease resistance protein rga3-like isoform x1	Coiled coil, Leucine rich repeats, NB-ARC	0	−0.74
INNBLQX01A9IWX	Disease resistance protein rga3-like isoform x1	Coiled coil, Leucine rich repeats, NB-ARC	0	−2.32
pm_c12557	Disease resistance protein rga3-like isoform x1	Coiled coil, Leucine rich repeats, NB-ARC	0.42	0.26
INNBLQX01BA29P	Disease resistance protein rga4-like	Coiled coil, Leucine rich repeats, NB-ARC	−1.00	0
INNBLQX01DD3SZ	Disease resistance protein rga4	Coiled coil, Leucine rich repeats, NB-ARC	0.00	0.26
INNBLQX01A9IWX	Disease resistance protein rga3-like isoform x1	Coiled coil, Leucine rich repeats, NB-ARC	−2.00	0
pm_c45828	Disease resistance protein rga1-like	Leucine rich repeats, NB-ARC and P-loop NTPase	1.00	0
pm_c26661	lz-nbs-lrr class rga	Leucine rich repeats, NB-ARC and P-loop NTPase	−1.58	−0.74
INNBLQX02JN1C8	Leucine-rich repeat-containing protein	Recognize and transmit pathogen-derived signals	3.17	−0.74
pm_c20365	nbs-lrr disease resistance protein homolog	Recognize and transmit pathogen-derived signals	1.58	0
pm_c12696	nbs-lrr class disease resistance protein	Recognize and transmit pathogen-derived signals	0.58	0
pm_c28200	nbs resistance partial	Recognize and transmit pathogen-derived signals	1.00	0
pm_c19586	nbs-lrr type resistance protein	Recognize and transmit pathogen-derived signals	1.32	0.26
pm_c27783	Leucine-rich repeat family protein	Recognize and transmit pathogen-derived signals	1.00	0
INNBLQX01ECXST	Probable disease resistance protein rf45-like	P loop and NB-ARC	1.58	0
pm_c30156	npr disease resistance protein	Ankyrin_rpt-contain_dom., NONEXPRESSOR OF PR GENES	−1.00	0
pm_c8238	f-box lrr-repeat protein 13-like	F-box/LRR-repeat protein 13-like, regulate salicylic acid-dependent gene expression during systemic acquired resistance.	0.81	0
INNBLQX01A2WWG	f-box lrr-repeat protein 3-like	F-box/LRR-repeat protein 13-like, regulate salicylic acid-dependent gene expression during systemic acquired resistance	0.58	0.26
pm_c26024	mlo-like protein 1-like	Seven transmembrane domains and calmodulin binding domain	2.70	1.26
INNBLQX02G8SE7	mlo-like protein 14	Seven transmembrane domains and calmodulin binding domain	1.58	−0.74
pm_c17303	nb-arc domain containing expressed	NB, ARC1, and ARC2, regulate activity of the resistance protein	1.00	0.26
INNBLQX01ADNB1	nb-arc domain-containing protein	NB, ARC1, and ARC2, regulate activity of the resistance protein	1.32	0
pm_c49493	Disease resistance protein at1g58400-like isoform x3	CC-NBS-LRR class family	0.57	0.12

*Positive sign indicate Up regulation, negative sign indicate Down regulation, n and 0 indicate no/undetectable transcript expression*.

**Figure 6:**
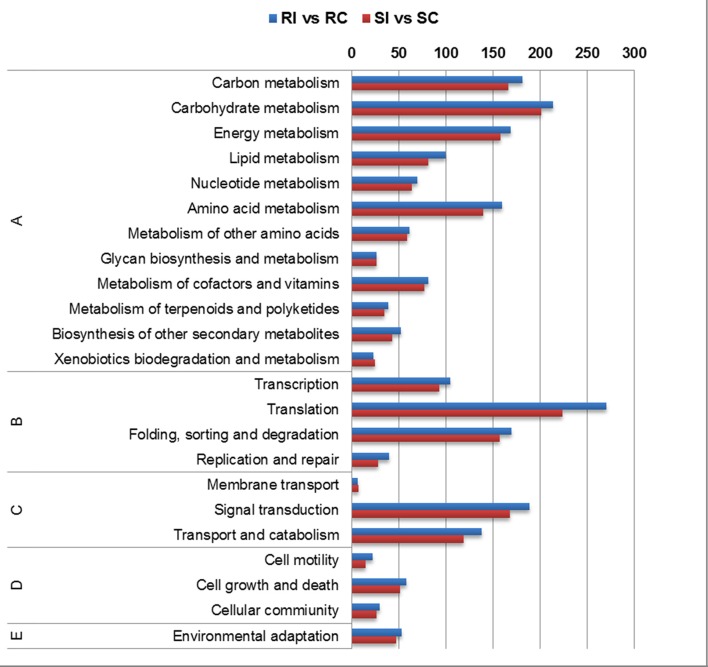
**Pathway enrichment of differentially expressed transcripts using KAAS annotation server (A) Metabolism; (B) Genetic Information Processing; (C) Environmental Information Processing; (D) Cellular Processes; (E) Organismal Systems**.

In KEGG annotation, the maximum transcripts were ascribed to carbohydrate, energy metabolism followed by amino acid metabolism among the primary metabolic pathways (Figure 6). Secondary metabolite pathway transcripts were ascertained in higher number in RI/RC. Transcripts for signal transduction pathway were higher in the RI/RC than SI/SC. Up-regulated pathways in the RI/RC included oxidative phosphorylation (ko00190), phenylalanine metabolism (ko00360), phenylalanine tyrosine tryptophan metabolism (ko00400), phenylpropanoid (ko00940), ubiquitin mediated proteolysis (ko04120), MAPK signaling (ko04010), cAMP signaling (ko04024), plant hormone signal transduction (ko04075), and plant pathogen interaction (ko04626).

**Figure 7:**
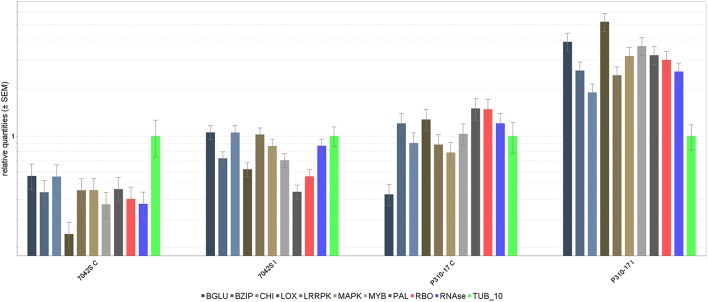
**Log2 fold differential gene expression of transcripts through Real- time PCR**. Bars on top show the standard deviation of three biological replications. Expression normalized with endogenous gene: Tubulin. 1. β Glucanase, 2. Basal zipper protein (BZIP), 3. Chitinase (CHI), 4. Lipoxygenase (LOX), 5. Leucine rich repeat protein kinase (LRRPK), 6. Mitogen activated protein kinase (MAPK), 7. MYB, 8. Phenylalanine ammonia lyase, (PAL) 9. Respiratory burst oxidase (RBO), 10. Ribonuclease (RNase).

**Figure 8:**
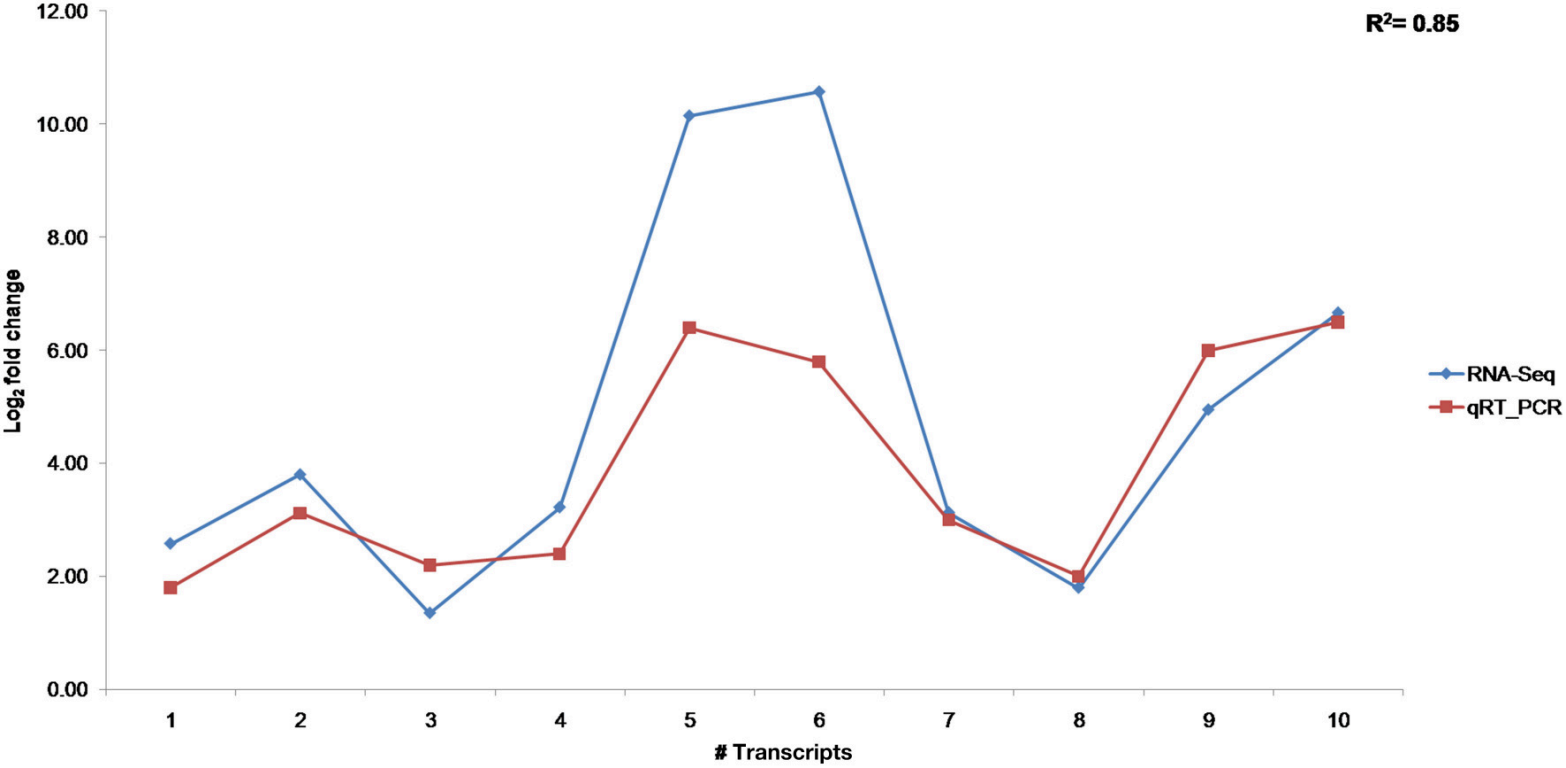
**Correlation of transcript expression between RNA-seq and qRT-PCR**.

The 10 transcripts validated through qRT-PCR represented functional classes of transcripts involved in plant defense mechanism like pathogenesis related proteins (PR), transcription factors, signaling and secondary metabolite pathways. The tubulin transcript, Tub_10 was ranked first according to its stability and used for normalizing relative gene expression of transcripts (Supplementary Table 4). Differential expression of transcripts was observed in RI and SI along with their respective controls (Figure 7). The lipoxygenase, MYB, and MAPK were among the highly up regulated transcripts in RI compared to SI. Up regulation of the defense related transcripts was also observed in SI but with lower expression than that in RI. Comparison of transcript expression levels between transcriptome data and qRT-PCR depicted positive correlation although the values for log2 fold change did not exactly match but remained consistent in expression (Figure 8).

## Discussion

The 454 Roche sequencing has been extensively employed for transcriptome sequencing and is well-suitable for both model and non-model plants with better performance than conventional methods of gene expression (Chen et al., 2013). In the present study, the sequencing throughput (684.97 Mb) and average length (433.99 bp) was consistent with the characteristic throughput and moderate read length of 454 GS FLX Titanium. It is very crucial to obtain cleaned, high-quality reads for further processing toward assembling, analysis of gene expression and hence, pre-processing was carried out on the raw sequencing reads (Martin and Wang, 2011; Vijay et al., 2013). Pre-processing removed 18% of the raw reads in the form of low complexity, low length, duplicate, and rRNA reads. To get a non-redundant data, duplicate reads were removed before assembling the reads into transcripts but were employed for back mapping while analyzing gene expression to decipher the level of transcript expression. We presented much attention to assembling the reads as it forms an essential view to determine how best to evaluate assemblies, particularly in light of the variety of options available and absence of reference genome (O'Neil and Emrich, 2013). For optimal generation of transcripts, it becomes compelling to compare more than one assembler for assembling the sequencing reads (Kumar and Blaxter, 2010). From the assembly comparisons of pearl millet transcriptome, it could be inferred that the assembler, MIRA performed well as compared to other three assemblers for the 454 data. This corroborates with the earlier studies wherein MIRA generated the maximum number of contigs, largest contig size and depicted the maximum similarity with relative species proteome (Garg et al., 2011; Guimaraes et al., 2012; Zhou et al., 2012). Appropriate usage of sequencing data was reflected as MIRA emanated in the maximum number of reads utilized for assembling. The assembly assessment forms a critical step for annotating and analyzing the transcriptome data and depends on the individual data sets and species in consideration (Garg et al., 2011). In addition, studies on transcriptome sequencing without reference genome stressed on assembly optimization step for better assembly (Chen et al., 2013). Generally, Newbler has been considered as the choice for assembling sequences generated from 454 Pyro sequencer and most of the previous studies on 454 transcriptome sequencing employed exclusively one assembler without any optimization (Kumar and Blaxter, 2010; Garg et al., 2011; Chen et al., 2013). Although, the N50 value was less for MIRA, N50 value has more significance in context of genome sequencing data than the transcriptome data. Moreover, the crucial aspect in transcriptome is number of relevant transcripts having meaningful annotation or function. The proteome coverage reflected the quality and significance of transcript formation for selection of best suitable assembly for pearl millet reads (O'Neil and Emrich, 2013). The length of the transcripts was also better represented in MIRA assembly resulting in 78.21% of transcripts having length more than 500 bp. The coverage of expressed transcripts compared with the closely related and completely sequenced crop, *S. italica* excogitated with 47.94% of transcripts having more than 80% coverage. The data is also supported by evidences of chromosomal relationships between pearl millet, foxtail millet, sorghum, and rice (Devos et al., 2000; Zhang et al., 2012). Also, in blast hit, the maximum number of pearl millet transcripts showed blast hits with foxtail millet which may point toward their ancestry and conservation of gene sequences within the Poacae family (Devos et al., 2000; Lata and Prasad, 2013). Blast hits with related oomycete species demonstrated the involvement of downy mildew transcripts in compatible interaction with host (Fawke et al., 2015). It depicted dual transcriptome wherein in compatible reaction the host and pathogen sequences were obtained (Kawahara et al., 2012; Westermann et al., 2012; Schulze et al., 2015). Withal, genome sequence information is not available for *S. graminicola*, homology of downy mildew transcripts involved in virulence or pathogenicity with other oomycetes species annotated in pathogen host interaction database was observed as avirulence determinant, reduced virulence, and unaffected pathogenicity but no further in detail analysis was performed (Jiang and Tyler, 2012).

### Gene for gene interaction

A compatible interaction between pearl millet and downy mildew culminates into a vegetative structure instead of reproductive structure i.e., panicle, drastically bringing down its productivity. The invasion of pathogen commences at an early stage followed by morphological changes in case of compatible and incompatible interactions. Subsequent upon the infection of pearl millet seedlings with downy mildew pathogen, the appearance of brown streaks was comparatively early in resistant inoculated seedlings about eight hpi than in susceptible seedlings in which it appeared after 12–15 hpi. This can be attributed to the hypersensitive response (HR) which could have mounted as a local defense mechanism against the downy mildew pathogen (Heath, 2000). The HR has been already reported in pearl millet-downy mildew interaction (Shivakumar et al., 2003). Resistance in the form of early hypersensitive response has been recorded in relative oomycetes pathogens (Kamoun et al., 1999). There are reports published on pearl-millet downy mildew interaction describing the histological studies in downy mildew resistant genotype after inoculation, in susceptible genotype and also in case of induced resistance in resistant genotypes (Kumudini et al., 2001; Shivakumar et al., 2003; Prabhu et al., 2012).

The disease resistance or R genes are key players in defense responses involved in the gene for gene interaction i.e., the R-Avr recognition and play critical role in recognition and PAMP based response (Gururani et al., 2012). Transcript for SGT1 was highly up regulated in the resistant genotype upon infection. It has been well-established that the SGT1 complex along with HSP90 and RAR1 protein is responsible for regulation of defense responses (Spoel and Dong, 2012). Other defense related transcripts like rpp gene, *Hyaloperonospora parasitica* resistance genes identified in Arabidopsis-downy mildew interaction were also found to be expressed. Interestingly, homology can be observed between the genes expressed in plants in response to classes of downy mildew causing pathogens (Asai et al., 2014). Upregulation of antioxidant enzymes, respiratory burst oxidase and superoxide dismutase (SOD) lined up with the occurrence of HR (Babitha et al., 2002; Mahatma et al., 2011). The upregulation of respiratory burst oxidase in RI was also confirmed by qRT-PCR. Dodds et al. (2009) reviewed that oomycetes express host translocated effectors and the R-Avr interaction leads to the primary response of HR.

### Global resistance machinery

In the present study, involvement of PR proteins in pearl millet-downy mildew interaction corroborated with the earlier studies (Shivakumar et al., 2000; Prabhu et al., 2012). PR proteins are induced locally and also activated as a response of systemic acquired resistance (SAR), in the present study also, transcripts for classes of PR proteins were expressed differentially. Expression of PR proteins has also been reported in various plant-oomycetes interactions (Van Loon and Van Strien, 1999; Glazebook, 2005). Of greater interest was purothionin, which was highly up regulated in resistant inoculated than the susceptible one. Although the PR proteins showed up-regulation in the susceptible inoculated sample, its expression was less than that in the resistant one. The expression of thionin (a cysteine rich polypeptide), thaumatin, Bowman-Birk type trypsin inhibitor-like protein can be extrapolated to have its role in anti-downy mildew activity (Chilosi et al., 2000; Chandrashekhara et al., 2010; Perazzolli et al., 2012). Expression of PR proteins has also been reported in various plant-fungal interactions including grape-downy mildew, Arabidopsis-downy mildew, foxtail-powdery mildew (Wu et al., 2010; Caillaud et al., 2013; Weng et al., 2014; Li et al., 2015). The transcript for oxalate oxidase was up-regulated and it is reported to be involved in plant defense (Zhang et al., 2013). Recently, in Arabidopsis-downy mildew interaction, it has been demonstrated that a downy mildew effector (RxLR) suppressed PR1 expression in cells containing haustoria and attenuated the salicylic acid–triggered immunity by interacting with the host mediator complex (Caillaud et al., 2013). Thus, it might be deduced that these PR proteins work in concert in bestowing resistance to pearl millet upon downy mildew infection. Moreover, the expression level of representative transcripts for PR protein was in concurrence with expression pattern in real time PCR.

Various families of transcription factors (TFs) were upregulated upon infection (Olga et al., 2014). Higher number of MYB and WRKY transcription factors corroborated with the earlier studies on plant-pathogen interaction (Djami-Tchatchou et al., 2012; Ambawat et al., 2013; Weng et al., 2014). The expression of MYB TFs in pearl millet-downy mildew might also indicate its involvement in defense and in HR (Vailleau et al., 2002). It has been shown in barley-powdery mildew interaction that WRKY transcription factors (HvWRKY1 and HvWRKY2) activated immediately followed by Avr recognition suggesting role of WRKY (De Wit et al., 2009). The activation of Myb and WRKY TFs corroborated well with earlier studies in rice-smut, rice-blast, sorgum-sorghicola, grape-downy mildew (Bagnaresi et al., 2012; Kawahara et al., 2012; Yazawa et al., 2013; Chao et al., 2014). Furthermore, WRKY, MYB, bhlh and bZIP regulate and/or interact with phenylpropanoid pathway biosynthetic genes potentially involved in defense (Dixon et al., 2002). The high level of expression of the transcription factors, MYB and bhlh was also in line with the relatively high expression in qRT-PCR. The HSP TF was observed to be up regulated, extending the hypothesis that heat shock protein families might be expressed in pearl millet to stabilize the proteins and membranes and to assist in protein refolding under stress condition initiated by downy mildew fungus.

The up-regulation of several classes of protein kinases *viz*, wall associated kinases, calcium-dependent protein kinases, LRR receptor-like protein kinases, serine threonine protein kinases, lrr serine threonine protein kinases, MAPKs suggested a strong activation of signal transduction machinery mediated by Ca^2+^ permeable channels following positive regulation by phosphorylation of kinase domains of the protein kinases (Heath, 2000; Hammond-Kosack and Parker, 2003; White and Broadley, 2003; King et al., 2014). The relative up-regulation of MAPK upon inoculation through quantitative validation suggested its potential role as signal carrying mediator in pearl millet downy mildew interaction (Melvin et al., 2014).

The hydroxyproline rich glycoproteins (HRGPs) constitute one of the important structural components of the cell wall involved in defense against plant pathogens. The HGRPs and related proteins of its superfamily *viz*., extensins, and proline-rich proteins were expressed in the transcriptome and co-related with studies of Sujeeth et al. (2010, 2012) in pearl millet-downy mildew interaction. The transcript for polygalacturonase inhibitor protein (PGIP), cell wall glycoprotein was expressed in the resistant genotype upon inoculation which corroborated with the previous reports of Prabhu et al. (2012) in pearl millet-downy mildew and that of Wu et al. (2010) in grape-downy mildew interaction. The PGIPs, concerned as defense proteins comprise of an extra cytoplasmic leucine rich repeats (eLRR) that specifically bind and inhibit fungal polygalacturonases, thus preventing cell wall invasion of fungus into host tissue (Di Matteo et al., 2006).

The plant defense responses are multi component in nature. With regards to systemic acquired resistance, salicylic acid is principally responsible for local and systemic responses (Kachroo and Robin, 2013). Recently, study of SAR in Arabidopsis suggested that a cluster of genes including signaling, secretory, and transducing compounds are up-regulated (Gruner et al., 2013). The rate limiting enzyme, PAL was up-regulated in RI than that in SI and also affirmed with the upregulation in RI through qRT-PCR. The transcription factors mainly, WRKY, MYB, and bHLH are mainly involved in the regulation of phenyl propanoid pathway and PR proteins (Dangl and Jones, 2001; Dixon et al., 2002). Significantly higher number of transcripts belonging to phenylalanine metabolic pathway was consistent with the activation SAR in pearl millet as a defense mechanism toward downy mildew. Moreover, it has been well-established from the previous studies on plant-oomycetes interaction that phenylalanine metabolic pathway is chiefly activated and SAR provides broad-spectrum resistance against oomycetes in addition to conferring immune memory (Naoumkina et al., 2010; Orłowska et al., 2012; Kachroo and Robin, 2013; Qiu et al., 2015; Serba et al., 2015).

It can be inferred from the results that defense mechanism in the host, pearl millet in response to downy mildew could be activated in the form of hypersensitive response carried by signal transducing molecules like lrr/serine-threonine/MAP kinases followed by the activation of the pathogenesis related proteins and phenylpropanoid pathway enzymes as a part of systemic acquired resistance. Thus, through transcriptome sequencing of downy mildew resistant and susceptible genotypes, we have comprehensively presented a snapshot of differential gene expression occurring in the cell at the time of downy mildew infection. The transcripts responsible for defense resistance can be characterized further through candidate gene based approach for identification of QTL for resistance breeding. Engineering pearl millet to over-express the enzymes that were up-regulated in the resistant genotype or genome editing would be a novel and effective strategy for enhancing resistance against downy mildew pathogen.

## Author contributions

YS and CJ conceived, planned and designed the study. KK, HZ, and TB carried work, analyzed, interpreted data, and prepared the manuscript. MP, HZ executed the data analysis. SK, RF, YS, and SN critically revised and edited the manuscript.

## Funding

The funds for research work was granted by Centre of Excellence in Agricultural Biotechnology, Anand Agricultural University, Anand, Gujarat (Grant number: Budget Head 12011).

### Conflict of interest statement

The authors declare that the research was conducted in the absence of any commercial or financial relationships that could be construed as a potential conflict of interest.

## Supplementary material

Supplementary Figure 1 **Schematic representation of workflow**.

Supplementary Figure 2 **Length wise distribution of pearl millet high quality reads**.

Supplementary Table 1 **List of primers of transcripts used for qRT-PCR**.

Supplementary Table 2 **Assembly assessment by BlastX with *Setaria italica* (Foxtail millet) proteome**.

Supplementary Table 3 **Annotation of pearl millet transcripts assembled by MIRA (XLSX)**.

Supplementary Table 4 **Evaluation of endogenous reference genes in pearl millet under downy mildew stress using RefFinder**.
