# Supplementary figures and images for: De novo Transcriptome Sequencing to Dissect Candidate Genes Associated with Pearl Millet-Downy Mildew (Sclerospora graminicola Sacc.) Interaction
Source: Front Plant Sci. 2016 Jun 22;7:847. doi: 10.3389/fpls.2016.00847 (PMC4916200; doi:10.3389/fpls.2016.00847)

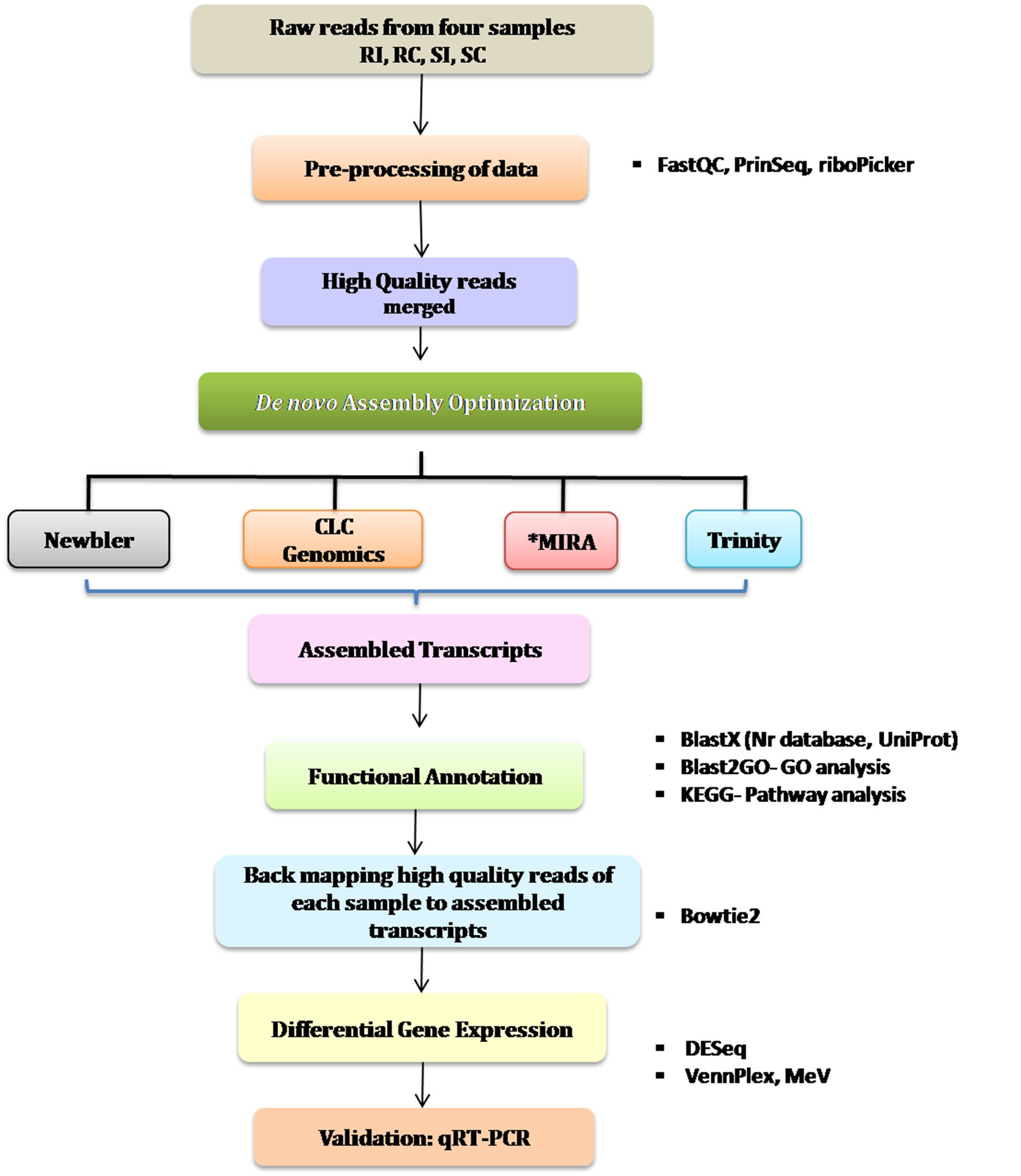

Supplement: Supplementary Figure 1 — Schematic representation of workflow. [file Image1.TIF]

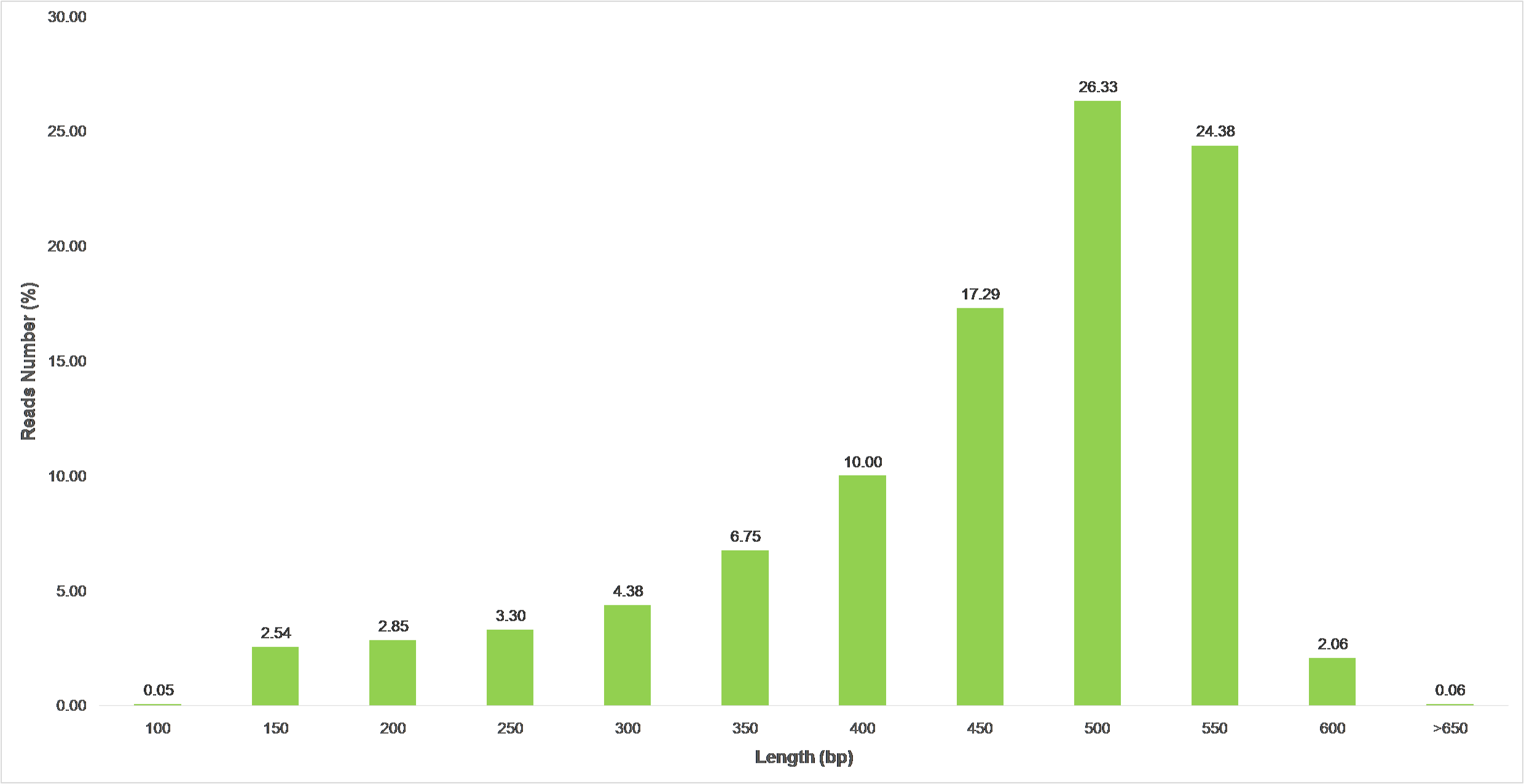

Supplement: Supplementary Figure 2 — Length wise distribution of pearl millet high quality reads. [file Image2.TIF]
