# Supplementary material for: De novo Transcriptome Sequencing to Dissect Candidate Genes Associated with Pearl Millet-Downy Mildew (Sclerospora graminicola Sacc.) Interaction
Source: Front Plant Sci. 2016 Jun 22;7:847. doi: 10.3389/fpls.2016.00847 (PMC4916200; doi:10.3389/fpls.2016.00847)
Supplement: Supplementary Table 1 — List of primers of transcripts used for qRT-PCR. [file Table1.DOCX]

**Supplementary Table 1:** **List of primers of transcripts used for qRT-PCR**

| **Sr. No.** | **Identifier of transcript in assembly /Name** | **Primer name** | **Primers sequences (5'-3')** | **Product Size** |
| --- | --- | --- | --- | --- |
|  | **Endogenous genes** | | |  |
| 1 | α-Tubulin transcript (pm_rep_c1485) | Tub_10 | GAAAGGCCAACCTACACCAA//ATGCGTGGGTATGGAACAAG | 138 |
| 2 | β-Tubulin transcript (pm_rep_c1485) | Tub_96 | AGCTCATCGACTCCGTCCT//TGGGTACTCCTCACGGATCTT | 149 |
| 3 | 18S (Ramineni et al., 2014*) | 18S | ATGCGCTCCTGGCCTTACT//TCATTACTCCGATCCCGAAG | 150 |
| 4 | Actin (*Setaria italica* actin) | Actin | AGGCAGAATCAGTGCCAAAC//CGGTTGTTTTAGCTGGTGGA | 160 |
|  | **Transcription factors** | | |  |
| 5 | MYB (pm_c9711) | MYB | CATGGAAGGGATATGTGCTTCT//CTGCTTTGTTCAATATGGTGGA | 152 |
| 6 | Basic leucine zipper (pm_c32092) | bZIP | AGTGCAAACAACTGAAAGTGGA//TAATTTGGTGGTGCATTGTAGC | 153 |
|  | **Defence related enzymes** | | |  |
| 7 | Lipoxygenase (pm_c12917) | LOX | CCGATCCAAGTAAGAAAAACGA//TCGCTAGGACAATTCAAAGATG | 158 |
| 8 | Phenylalanine ammonia lyase (pm_rep_c1156) | PAL | GATTGTCAAGCTGCTCAACG//GGCTACTGCCAAAGAGTTCG | 136 |
| 9 | Respiratory burst oxidase (pm_c2220) | RBO | GCTGATGGTCAGATCACTGAAG//GGCCGCAAAATCATTAACTATC | 152 |
|  | **Pathogenesis-related proteins** | | |  |
| 10 | β-1,3 Glucanase (pm_c17238) | BGLU | GGCATTGACCACTACAACAAAG//ATCTCAATGTAGCCGAGGTTGT | 150 |
| 11 | Ribonuclease (pm_c1460) | RNase | CAACGGTCAGAGGGAGTAAAGA//CAAGGTTACAAACGCGCTAAA | 145 |
| 12 | Chitinase (pm_c6348) | CHI | GTAGAACTGCACCCACACGAA//  GTCAAGGACATGGGCAAGAAC | 148 |
|  | **Signaling molecules** | | |  |
| 12 | Leucine rich repeat protein kinase (pm_c14579) | LRRPK | ACTGTAAGCAAACCAGTGAGCA//AACTGTACTTGACGCCCCATAC | 147 |
| 13 | Mitogen activated protein kinase (pm_c12463) | MAPK | TTGCCAGTGTATTGTGTGAGTG//GAATATGAAAATGTTGCGCTGT | 140 |

*Ramineni, R., Sadumpati, V., Khareedu, V. R., Vudem, D.R. (2014) Transgenic pearl millet male fertility restorer line (ICMP451) and hybrid (ICMH451) expressing *Brassica juncea* Nonexpressor of Pathogenesis Related Genes 1 (*BjNPR1*) exhibit resistance to downy mildew disease. *PLoS ONE* ***9(3): e90839***.
