# Supplementary material for: De novo Transcriptome Sequencing to Dissect Candidate Genes Associated with Pearl Millet-Downy Mildew (Sclerospora graminicola Sacc.) Interaction
Source: Front Plant Sci. 2016 Jun 22;7:847. doi: 10.3389/fpls.2016.00847 (PMC4916200; doi:10.3389/fpls.2016.00847)
Supplement: Supplementary Table 2 — Assembly assessment by BlastX with Setaria italica (Foxtail millet) proteome. [file Table2.DOCX]

**Supplementary Table 2: Assembly assessment by BlastX with *Setaria italica* (Foxtail millet) proteome**

| **Assembly** | **Newbler** | | **CLC** | | **MIRA** | | **Trinity** | |
| --- | --- | --- | --- | --- | --- | --- | --- | --- |
| Length coverage  bin (%) | count_ in_bin | >bin_below | count_ in_bin | >bin_below | count_ in_bin | >bin_below | count_ in_bin | >bin_below |
| 100 | 2258 | 2258 | 2111 | 2111 | 3141 | 3141 | 2849 | 2849 |
| 90 | 860 | 3118 | 974 | 3085 | 1149 | 4290 | 1156 | 4005 |
| 80 | 760 | 3878 | 1046 | 4131 | 1116 | 5406 | 1040 | 5045 |
| 70 | 832 | 4710 | 1234 | 5365 | 1312 | 6718 | 1147 | 6192 |
| 60 | 867 | 5577 | 1440 | 6805 | 1507 | 8225 | 1309 | 7501 |
| 50 | 928 | 6505 | 1794 | 8599 | 1804 | 10029 | 1553 | 9054 |
| 40 | 910 | 7415 | 1976 | 10575 | 2019 | 12048 | 1589 | 10643 |
| 30 | 884 | 8299 | 2007 | 12582 | 2030 | 14078 | 1554 | 12197 |
| 20 | 885 | 9184 | 1946 | 14528 | 2108 | 16186 | 1537 | 13734 |
| 10 | 0 | 9184 | 0 | 14528 | 0 | 16186 | 0 | 13734 |
| 0 | 0 | 9184 | 0 | 14528 | 0 | 16186 | 0 | 13734 |

Count_in_bin: Count of transcripts in bin for each assembler

>bin_below: Cumulative count of transcripts at or above bin level for each assembler
