# Supplementary material for: De novo Transcriptome Sequencing to Dissect Candidate Genes Associated with Pearl Millet-Downy Mildew (Sclerospora graminicola Sacc.) Interaction
Source: Front Plant Sci. 2016 Jun 22;7:847. doi: 10.3389/fpls.2016.00847 (PMC4916200; doi:10.3389/fpls.2016.00847)
Supplement: Supplementary Table 4 — Evaluation of endogenous reference genes in pearl millet under downy mildew stress using RefFinder. [file Table4.docx]

**Supplementary Table 4: Evaluation of endogenous reference genes in pearl millet under downy mildew stress using RefFinder**

| **Name of genes** | **Delta C_T_** | **Best keeper** | **Norm finder** | **geNorm** | **Comprehensive ranking** |
| --- | --- | --- | --- | --- | --- |
|  | Average StdDev | Standard Deviation | Stability value | Stability value | Geomean  ranking value |
| TUB_10 | 0.89 (1) | 0.29 (1) | 0.259 (1) | 0.562 (1) | 1.00 (1) |
| 18S | 1.02 (2) | 0.61 (3) | 0.721 (2) | 0.562 (1) | 1.86 (2) |
| ACTIN | 1.15 (3) | 0.44 (2) | 0.901 (3) | 0.926 (2) | 2.71 (3) |
| TUB_96 | 1.21 (4) | 0.70 (4) | 1.003 (4) | 1.069 (3) | 4.00 (4) |
